# Efficacy and Insights from an Extensive Series of Cytoreductive Surgery for Peritoneal Neoplasms: A High-Volume Single-Center Experience

**DOI:** 10.3390/cancers16244229

**Published:** 2024-12-19

**Authors:** Matteo Aulicino, Francesco Santullo, Giorgio D’Annibale, Carlo Abatini, Miriam Attalla El Halabieh, Cecilia Orsini, Lorenzo Barberis, Luca D’Agostino, Ilaria Tersigni, Fiammetta Pacelli, Claudio Lodoli, Andrea Di Giorgio, Federica Ferracci, Fabio Pacelli

**Affiliations:** 1 General Surgery Department, Università Cattolica del Sacro Cuore, 00168 Rome, Italy; matteo.aulicino01@icatt.it (M.A.); cecilia.orsini01@icatt.it (C.O.); lorenzo.barberis01@icatt.it (L.B.); lucadago93@gmail.com (L.D.); ilariatersigni@yahoo.it (I.T.); federica.ferracci@guest.policlinicogemelli.it (F.F.); fabio.pacelli@policlinicogemelli.it (F.P.); 2 Surgical Unit of Peritoneum and Retroperitoneum Surgery, Fondazione Policlinico Universitario Agostino Gemelli IRCCS, 00168 Rome, Italy; carlo.abatini@guest.policlinicogemelli.it (C.A.); miriam.attallaelhalabieh@guest.policlinicogemelli.it (M.A.E.H.); claudio.lodoli@policlinicogemelli.it (C.L.); andrea.digiorgio@policlinicogemelli.it (A.D.G.); 3 Department of Surgical and Medical Sciences and Translational Medicine, Sant ’Andrea University Hospital, Sapienza University of Rome, 00185 Rome, Italy; fiammetta.pacelli@gmail.com

**Keywords:** surgical oncology, CRS, cytoreductive surgery, HIPEC, PIPAC, peritoneal carcinomatosis, oncological outcomes, surgical complications

## Abstract

**Background:** Advances in cytoreductive surgery (CRS) combined with hyperthermic intraperitoneal chemotherapy (HIPEC) and pressurized intraperitoneal aerosol chemotherapy (PIPAC) have improved outcomes for selected patients with peritoneal surface malignancies (PSMs). **Methods:** This retrospective study analyzed 743 PSM patients treated at Fondazione Policlinico Universitario Agostino Gemelli from January 2016 to February 2024. The primary aim was to assess median overall survival (mOS), median disease-free survival (mDFS), and median progression-free survival (mPFS) stratified by tumor origin. Secondary outcomes examined the role of diagnostic laparoscopy in the management of PSMs and intra- and postoperative complications’ rates. **Results:** A total of 1113 procedures were performed: 389 CRS, 370 PIPAC, and 354 diagnostic laparoscopies. Colorectal cancer was the predominant indication for CRS (52.4%), with a mOS of 52 months and mDFS of 22 months. Patients affected by gastric cancer undergoing CRS had a mOS of 18 months and a mDFS of 13 months, while PIPAC yielded a mOS of 9 months and a mPFS of 4 months. Among patients with pseudomyxoma peritonei undergoing CRS, the 5-year DFS rate was 64.1%, and OS rate was 89%. Patients affected by mesothelioma and treated with CRS exhibited a median OS of 43 months and a DFS of 26 months. Pancreatic and hepatobiliary cancers were treated with PIPAC, with a respective mOS of 12 and 8 months. Postoperative complications occurred in 12.6% of CRS, 3.2% of PIPAC, and 1.7% of diagnostic laparoscopies. High peritoneal cancer index (PCI), gastric resection, and blood loss over 500 mL were identified as risk factors for major complications in a multivariate analysis. **Conclusions:** Developing a highly experienced multidisciplinary team is crucial for delivering tailored treatment strategies which aim to achieve optimal oncological outcomes while preserving patients’ quality of life.

## Simple Summary

Cytoreductive surgery and intraperitoneal chemotherapy (HIPEC or PIPAC) are effective treatment options for peritoneal carcinomatosis from various primary diseases. An accurate pre-operative assessment and multidisciplinary evaluations are crucial in identifying the most suitable treatment approaches. This study aims to present a case series from a high-volume center to contribute valuable data to the existing literature on peritoneal carcinomatosis managing.

## 1. Introduction

Peritoneal surface malignancies (PSM) are a heterogeneous group of tumors, including primary peritoneal neoplasms, such as mesothelioma, and secondary metastatic spreading sites originating from various intra-abdominal organs, such as the colon, stomach, ovaries, pancreas, or hepatobiliary tract. Pseudomyxoma peritonei is a distinct clinical entity, typically arising from appendiceal mucinous neoplasms, characterized by mucinous intraperitoneal dissemination.

The management of PSMs remains a significant challenge for surgeons and oncologists worldwide. Historically considered an almost terminal condition, nowadays substantial advancements have transformed the landscape of peritoneal malignancies, also enabling radical treatment approaches in some highly selected cases [1,2,3].

To overcome the so-called peritoneum–plasma barrier, the idea to integrate intraperitoneal drugs with systemic chemotherapy has determined the development of innovative surgical strategies [4,5].

For patients with limited peritoneal disease, cytoreductive surgery (CRS) eventually combined with hyperthermic intraperitoneal chemotherapy (HIPEC) offers a potentially curative approach [6,7]. This combination aims to eradicate macroscopic and microscopic tumor residues, leveraging the enhanced penetration of chemotherapeutic agents in residual disease. Despite historical concerns regarding the high surgical complexity associated with high morbidity and mortality rates, complete cytoreduction can result in long-term survival for selected patients, depending on tumor histology [8,9,10,11].

On the other hand, a newer therapeutic option for PSMs is represented by pressurized intraperitoneal aerosol chemotherapy (PIPAC), which delivers chemotherapeutic agents directly to peritoneal implants through a minimally invasive laparoscopic approach. PIPAC seeks to increase the local drug concentration while minimizing systemic toxicity compared to traditional intravenous chemotherapy [12]. Although PIPAC has demonstrated safety, tolerability, and efficacy in reducing peritoneal tumor burden across different cancer types, its impact on overall survival remains unclear and under investigation [13,14].

Nowadays, even if intraperitoneal treatments have become integral part of the clinical management of PSM, there are still ongoing debates regarding various aspects of these treatments: indications, patient’s selection criteria, and standardization of techniques.

The aim of this article is to assess the outcomes achieved in a high-volume tertiary center focused on peritoneal surface malignancy management.

## 2. Materials and Methods

### 2.1. Study Design

This retrospective study included a series of 743 patients with peritoneal surface malignances (PSMs) treated with cytoreductive surgery, PIPAC, or diagnostic laparoscopy in the Peritoneum and Retroperitoneum Surgical Unit at the Fondazione Policlinico Universitario A. Gemelli IRCCS from January 2016 to February 2024. The inclusion criteria were age >18 years and presence of primary peritoneal tumor (malignant or multi-cystic mesothelioma) or peritoneal carcinomatosis from different primary tumors (colorectal, gastric, appendiceal, pancreatic, hepatobiliary, or other origins). The primary endpoint of this study was to determine, based on the primary tumor type, the overall survival (OS) and disease-free survival (DFS) following cytoreductive surgery, including median survival times and 1-year, 3-year, and 5-year survival rates. Moreover, we evaluate OS and progression-free survival (PFS) from the first pressurized intraperitoneal aerosol chemotherapy (PIPAC), reporting median survival times and 1-year, 2-year, and 3-year survival rates. We also aimed to evaluate the impact of diagnostic laparoscopy on the proper management of patients with peritoneal surface malignancies (PSMs) in terms of reducing the incidence of unnecessary laparotomies. The secondary endpoint was to evaluate intraoperative and postoperative outcomes based on the surgical procedure performed. A univariate and multivariate analysis was conducted to assess factors influencing the occurrence of major postoperative complications in cytoreductive surgery. All patients provided written informed consent and were enrolled in a follow-up program. The relapse and survival data were collected and stored in a prospectively maintained database.

### 2.2. Study Population and Management

All patients enrolled in the study underwent a strict pre-operative workout. The primary disease was evaluated by endoscopic (EGDS, RSCS, EUS) and radiological examination (CT, MRI, PET-CT). The initial burden of peritoneal disease was assessed, whenever possible, using diagnostic laparoscopy, and a peritoneal carcinomatosis index (PCI) score was recorded for each patient [15]. The policy was to offer CRS + HIPEC in case of peritoneal primary disease and in case of secondary peritoneal malignances from colorectal adenocarcinoma or gastric cancer without extra-abdominal metastases and a potentially achievable CC:0. For pseudomyxoma peritonei and multi-cystic mesothelioma, a CC:1 result was also considered acceptable for CRS, due to a lower biological aggressiveness and to guarantee an improvement in the quality of life. HIPEC after CRS was not administered in case of incomplete cytoreduction or in patients with poor clinical conditions after extensive surgery with multiple visceral resections. In accordance with current global practices and scientific evidence in PSM treatment, PIPAC was indicated in combination with systemic chemotherapy (bimodal treatment) for patients who were not eligible for cytoreductive surgery (CRS) due to disease extent or primary tumor type (pancreatic or hepatobiliary cancer). Eligibility criteria for PIPAC included a small size and volume of PM (smaller than 2 cm and/or not confluent, as large carcinosis nodules would not allow optimal drug penetration), absence of abdominal or extra-abdominal disease with oncological progression, no extensive abdominal adhesions, and no bowel obstruction due to PM. To all patients who were not eligible either for CRS or PIPAC, systemic chemotherapy was offered. CRS was performed using the technique described by Sugarbaker [5]. PIPAC was performed according to current recommendations and safety protocols [16,17]. Follow-up protocol included physical examinations, measurement of tumor marker levels (CEA, CA 125, CA 19.9), and regular radiological imaging (CT thorax and abdomen scheduled every 3–6 months). The follow-up schedule was tailored based on the primary tumor and in accordance with different chemotherapeutic regimens.

### 2.3. Data

OS was defined as the period from surgery to the date of death from any cause or censored at the date of the last follow-up for surviving patients. DFS was defined as the period from cytoreductive surgery to the date of documented recurrence. PFS was defined as the period from PIPAC to the date of documented progression of disease. Clinical variables collected included age, gender, body mass index (BMI), Eastern Cooperative Oncology Group (ECOG) Performance Status [18], American Society of Anaesthesiologists score (ASA), primary tumor, time of carcinomatosis onset, preoperative chemotherapy, and Peritoneal Cancer Index (PCI) score. Surgical intraoperative details included operative time, HIPEC or PIPAC drug used, the extent of peritonectomy in CRS, and the necessity of multi-visceral resections or concomitant procedures (gastric resections, hepatic resections, small bowel resections, splenectomy, hystero-annexectomy). Blood loss, number of anastomoses performed, and intraoperative complications were considered in the analysis. Postoperative complications were classified according to the Clavien–Dindo classification.

### 2.4. Statistics

Descriptive statistics were used to delineate patient’s surgical and pathological characteristics. Continuous variables were expressed as medians and ranges, while categorical variables were presented as frequencies and percentages of the total cohort. Survival analysis was conducted using the Kaplan–Meier method. Multivariable analyses were performed using multinomial logistic regression integrating with univariate *p*-values ≤ 0.1. A *p*-value ≤ 0.05 was considered statistically significant and confidence intervals (CI) were established at 95%. Statistical analyses and graphics were performed using IBM SPSS version 24.0 (IBM, Armonk, NY, USA) software for Windows and Excel v16.54.

## 3. Results

A total of 1113 surgical procedures were performed on 743 patients with PM from different primary tumors, specifically, 389 cytoreductions, 370 PIPACs, and 354 diagnostic laparoscopies. The baseline characteristics and surgical details of patients who underwent cytoreduction or PIPAC treatment are described in Table 1A and Table 1B, respectively. The long-term oncological outcomes for each pathology, based on the treatment performed, are evaluated in the following sections.

### 3.1. Colorectal Cancer Carcinomatosis

Colorectal cancer was the most frequent indication for CRS in our series, with 204 cases (52.4%). In 88 patients (43.1%), a synchronous onset of carcinoses was observed. The median surgical PCI was 6 (range 1–26). Complete cytoreduction (CC-0) was achieved in 188 patients (92.2%). HIPEC was performed in 178 patients (87.3%). In the first 28 patients (14.4%), oxaliplatin was used. Based on overall experience and the scientific literature, the subsequent 176 patients (86.3%) were treated with Mitomycin C. Mitomycin C was used at high dose (35 mg/mq) for 90 min. Major complications occurred in 20 patients (9.8%). With a median follow-up of 43 months, the median DFS was 22 months (1-year DFS of 80.9%, 3-year DFS of 22.2%, and 5-year DFS of 13.4%), and the median OS was 52 months (1-year OS of 96%, 3-year OS of 63.8%, and 5-year OS of 43.2%).

The indication for PIPAC in colorectal cancer was less frequent and was performed in 31 patients (16.9%) for an amount of 61 procedures (16.5%). The mean procedure number per patient was 1.9: 17 patients (54.8%) underwent only 1 PIPAC, 5 patients (16.1%) underwent 2 PIPACs, 3 patients (9.6%) underwent 3 PIPACs, 5 patients (16.1%) underwent 4 PIPACs, 1 patient (3.2%) underwent 5 PIPAs. Nine patients (29%) underwent more than three PIPAC procedures. The median PCI was 27. The intraperitoneal chemotherapeutic agent used was oxaliplatin 92 mg/mq. During PIPAC treatment, two patients (6.6%) showed a peritoneal disease regression to undergo CRS. The median follow-up was 18 months. Major complications occurred in 1 case (1.7%) The median PFS was 7 months (1-year PFS of 36%, 2-year PFS of 4%, and 3-year PFS of 4%), and the median OS was 16 months (1-year OS of 64.4%, 2-year OS of 27.6%, and 3-year OS of 27.6%).

**Figure 1:**
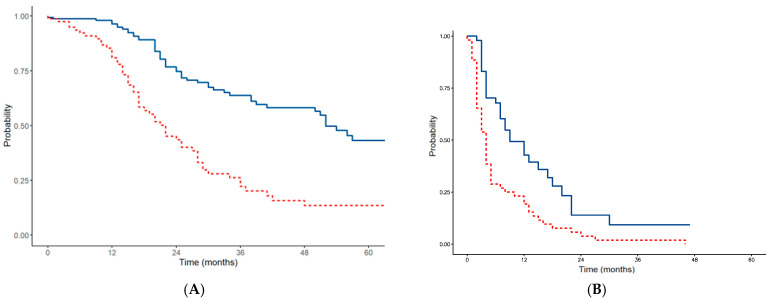
DFS/PFS (red line) and OS (blue line) of patients affected by colorectal cancer carcinomatosis treated with CRS (**A**) and PIPAC (**B**).

The DFS and OS of patients affected by colorectal cancer carcinomatosis are represented in the Kaplan–Meier curves in Figure 1A(CRS),B(PIPAC).

### 3.2. Gastric Cancer Carcinomatosis

Thirty-nine (10%) cytoreductions were performed in patients with gastric cancer and synchronous carcinomatosis. The median surgical PCI was 5.5 (range 1–14). Complete cytoreduction (CC-0) was achieved in 37 patients (94.9%). HIPEC was performed in all patients (100%), and the combination of Mitomycin C and cisplatin was always used as the chemotherapeutic agent. Major complications occurred in 6 patients (15.4%). The median follow-up was 24 months. The median DFS was 13 months (1-year DFS of 50.2%, 3-year DFS of 12.7%, and 5-year DFS of 0%), and the median OS was 18 months (1-year OS of 78.2%, 3-year OS of 28%, and 5-year OS of 18.7%).

Gastric cancer was the most frequent indication for PIPAC, with 144 procedures (38.9%) in 69 patients (37.7) The mean number of procedures per patient was 2.1. A total of 30 patients (43.5%) underwent 1 PIPAC, 19 patients (27.5%) underwent 2 PIPACs, 11 patients (15.9%) underwent 3 PIPACs, 6 patients (8.7%) underwent 4 PIPACs, 1 patient (1.4%) underwent 5 PIPACs, and 2 patients (2.9%) underwent 7 PIPACs. Twenty patients (29%) underwent more than three PIPAC procedures. The median PCI was 22. The intraperitoneal chemotherapeutic agent used was cisplatin 7.5mg/m and doxorubicin 1.5 mg/m2 in the first phase of our experience. As suggested by the results of the phase I trial by Tempfer in 2018, the recommended dose was increased to 10.5 mg/m2 for cisplatin and 2.1 mg/m2 for doxorubicin. The new protocol was adopted for all patients with PM from gastric cancer treated with PIPAC. During PIPAC treatment, 9 patients (13%) showed a peritoneal disease regression to undergo CRS. The median follow-up was 8 months. The median PFS was 4 months (1-year PFS of 19.2%, 2-year PFS of 3.8%, and 3-year PFS of 1.9%), and the median OS was 9 months (1-year OS of 42.7%, 2-year OS of 14%, and 3-year OS of 9.3%).

**Figure 2:**
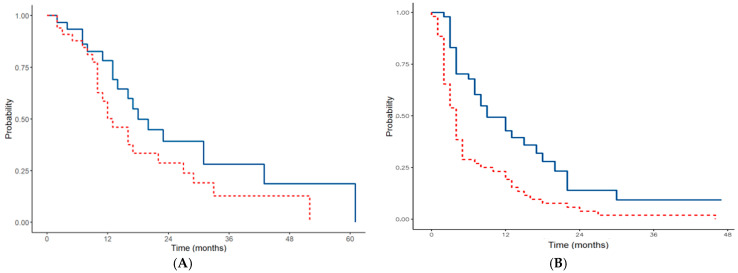
DFS/PFS (red line) and OS (blue line) of patients affected by gastric cancer carcinomatosis treated with CRS (**A**) and PIPAC (**B**).

The DFS and OS of patients affected by gastric cancer carcinomatosis are represented in the Kaplan–Meier curves in Figure 2A(CRS),B(PIPAC).

### 3.3. Pseudomyxoma Peritonei

The main treatment strategy for pseudomyxoma peritonei is CRS that was performed in 104 patients (26.7%). The median surgical PCI was 21.5 (range 1–33). Complete cytoreduction (CC-0) was achieved in 87 patients (83.7%). HIPEC was performed in 93%, and Mitomycin C was used as a chemotherapeutic agent. Major complications occurred in 18 patients (17.3%). The median follow-up was 62 months. The median overall survival and median DFS were not calculable. The 1-year DFS rate was 92.5%, 3-year DFS rate was 73.7%, and 5-year DFS rate was 64.1%. The 1-year OS rate was 100%, 3-year OS rate was 93.2%, and 5-year OS rate was 89%.

**Figure 3:**
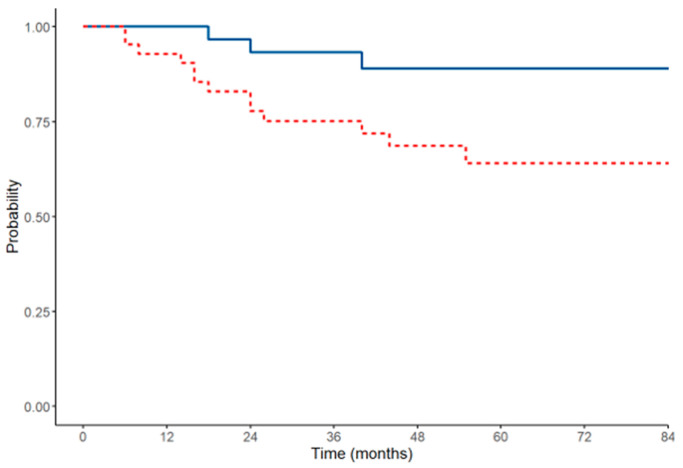
DFS (red line) and OS (blue line) of patients affected by pseudomyxoma peritonei treated with CRS.

The DFS and OS of patients treated with CRS are represented in the Kaplan–Meier curves in Figure 3.

Consistent with current scientific evidence and global indication on the treatment of pseudomyxoma peritonei, PIPAC has never been performed in patients affected by pseudomyxoma peritonei.

### 3.4. Mesothelioma

**Figure 4:**
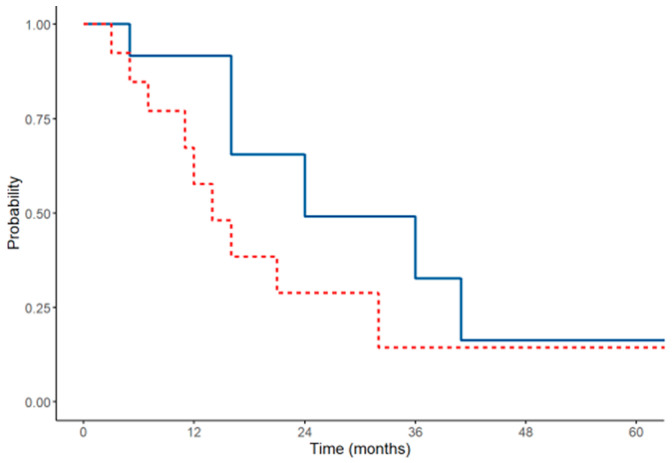
DFS (red line) and OS (blue line) of patients affected by pseudomyxoma peritonei treated with CRS.

Twenty-seven (6.8%) cytoreductions were performed in patients with mesothelioma. The median surgical PCI was 23 (range 1–31). Complete cytoreduction (CC-0) was achieved in 25 patients (92.6%). HIPEC was performed in 92%, and the combination of Mitomycin C and cisplatin was always used as the chemotherapeutic agent. Major complications occurred in 3 patients (11.1%). The median follow-up was 45 months. The median DFS was 26 months (1-year DFS of 81.3%, 3-year DFS of 36.4%, and 5-year DFS of 24.2%), and the median OS was 43 months (1-year OS of 94.1%, 3-year OS of 62.7%, and 5-year OS of 42.4%). The Kaplan–Meier curves in Figure 4 illustrate the DFS and OS of patients affected by mesothelioma who underwent CRS.

Similarly to pseudomyxoma, PIPAC treatments were not performed in patients diagnosed with mesothelioma.

### 3.5. Pancreatic Cancer and Hepatobiliary Cancer Carcinomatosis

In accordance with current global practices and scientific evidence, no cytoreductive surgery was performed in patients with carcinomatosis from pancreatic or hepatobiliary tumors.

Conversely, pancreatic cancer was the second most frequent indication for PIPAC, with 118 procedures (31.9%) performed in 56 patients (30.6%). The mean number of procedures per patient was 2.1. A total of 23 patients (41.1%) underwent 1 PIPAC, 16 patients (28.6%) underwent 2 PIPACs, 9 patients (16.1%) underwent 3 PIPACs, 6 patients (10.7%) underwent 4 PIPACs, 1 patient (1.8%) underwent 5 PIPACs, and 1 patient (1.8%) underwent 7 PIPACs. Seventeen patients (30.4%) underwent more than three PIPAC procedures. The median PCI was 8. The intraperitoneal chemotherapeutic agent used was cisplatin with doxorubicin or Nabpaclitaxel (abraxane) for those patients admitted to the Nab-pipac protocol. The median follow-up was 11 months. The median PFS was 4 months (1-year PFS of 17.4%, 2-year PFS of 0%, and 3-year PFS of 0%), and the median OS was 12 months (1-year OS of 48.9%, 2-year OS of 21.4%, and 3-year OS of 0%).

A total of 15 patients (8.2%) with carcinomatosis from hepatobiliary cancer underwent PIPAC, resulting In 27 procedures (7.3%). The mean number of procedures per patient was 1.8: nine patients (60%) underwent one PIPAC, three patients (20%) underwent two PIPACs, one patient (6.7%) underwent three PIPACs, one patient (6.7%) underwent four PIPACs, and one patient (6.7%) underwent five PIPACs. Three patients (20%) underwent more than three PIPAC procedures. The median PCI was 17. The intraperitoneal chemotherapeutic agent used was cisplatin with doxorubicin. The median follow-up was 7 months. The median PFS was 4 months (1-year PFS of 0%, 2-year PFS of 0%, and 3-year PFS of 0%), and the median OS was 8 months (1-year OS of 33.33, 2-year OS of 21.4%, and 3-year OS of 0%).

**Figure 5:**
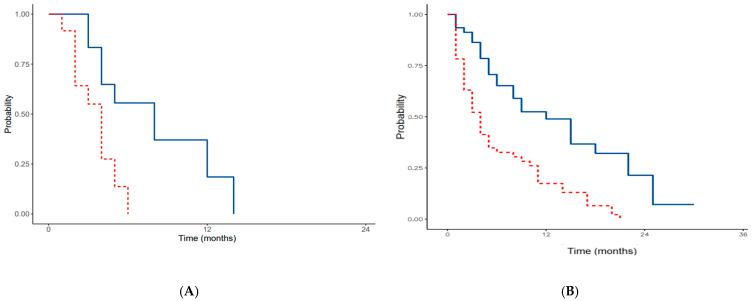
PFS (red line) and OS (blue line) of patients treated with PIPAC and affected by carcinomatoses from pancreatic cancer (**A**) and hepatobiliary cancer (**B**).

The DFS and OS of patients treated with PIPAC are represented in the Kaplan–Meier curve in Figure 5A(pancreatic cancer),B (hepatobiliary cancer).

### 3.6. Diagnostic Surgical Procedure

Out of 354 total procedures, 252 (71.2%) were performed as a preliminary step to cytoreduction. The remaining 102 laparoscopies were performed for diagnostic purposes in patients with radiological evidence of carcinomatosis with an occult primary tumor, or in patients not eligible for CRS, who were therefore referred for PIPAC treatment or chemotherapy.

**Figure 6:**
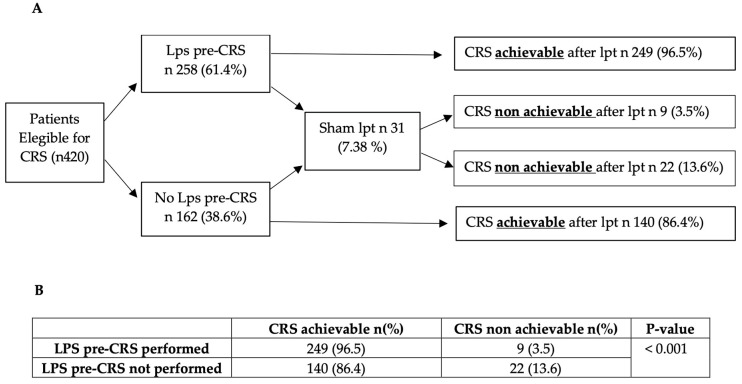
Flowchart of patients eligible for CRS (**A**). Incidence of unnecessary laparotomies, comparing patients with and without pre-CRS laparoscopy, analyzed via chi-square test (**B**).

As shown in Figure 6, out of 420 patients scheduled for CRS, 61.4% underwent pre-CRS laparoscopy, while 38.6% proceeded to CRS without laparoscopic staging. The overall incidence rate of sham laparotomies among all CRS patients was 7.38%. A statistically significant difference was observed between patients who had pre-CRS laparoscopy and those who hadn’t, with incidence of sham laparotomy’s rate rates among 3.5% and 13.6%, respectively (*p* < 0.001).

### 3.7. Intraoperative Complications in Treatment of Peritoneal Surface Malignancies

In line with the complexity of the procedure, operative times, blood loss, and intraoperative complications were higher in cytoreductive surgery (CRS). Of a total of 54 (13.9%) intraoperative complications during CRS, the most frequent were diaphragmatic laceration (7.5%) and bladder injury (2.8%), while the remaining complications occurred in less than 1% of cases. In PIPAC and diagnostic laparoscopy, the only complication observed was bowel perforation, with an incidence of 0.54% and 0.28%, respectively (Table 2). These conditions were treated with bowel resections, and no major complications occurred in the postoperative period.

### 3.8. Postoperative Complications in the Treatment of Peritoneal Surface Malignancies

Table 3 summarizes postoperative complications based on the surgical treatment performed. The incidence of major complications (grade ≥ 3) according to the Clavien–Dindo classification was 12.6% in CRS, 3.2% when PIPAC was performed, and 1.7% in case of diagnostic laparoscopy. The 30-day mortality was 1.3% and 1.1% in cytoreductive surgery and pressurized intraperitoneal aerosol chemotherapy (PIPAC), respectively. No deaths occurred during the hospital stay or within the first 30 days postoperatively in patients who underwent diagnostic laparoscopy.

A univariate analysis identified the following as risk factors for major postoperative complications: synchronous peritoneal carcinomatosis (OR = 2.047, 95% CI [1.013–4.134], *p* = 0.046), PCI (OR = 1.075, 95% CI [1.034–1.117], *p* = 0.0001), splenectomy (OR = 1.895, 95% CI [1.043–3.442], *p* = 0.036), gastric resection (OR = 2.430, 95% CI [1.180–5.005], *p* = 0.016), diaphragmatic peritonectomy (OR = 2.879, 95% CI [1.567–5.287], *p* = 0.001), pelvic peritonectomy (OR = 2.464, 95% CI [1.158–5.242], *p* = 0.019), blood loss > 500 mL (OR = 3.480, 95% CI [1.865–6.492], *p* = 0.019), and the number of anastomoses performed (OR = 1.579, 95% CI [1.103–2.257], *p* = 0.013). Conversely, colonic origin was found to be a protective factor (OR = 0.436, 95% CI [0.234–0.812], *p* = 0.009). Multivariate analysis confirmed PCI (OR = 1.059, 95%CI [1,014–1.107], *p* = 0.01), gastric resection (OR = 5.251, 95%CI [1.889–14.593], *p* = 0.001), and blood loss > 500 mL (OR = 4.286, 95%CI [1.780–10.317], *p* = 0.001) as independent factors that increase the risk of major complication (Table 4).

## 4. Discussion

The results reported from this study highlight that a multidisciplinary therapeutic approach can improve oncological outcomes for patients with peritoneal surface malignancies (PSMs). Guidelines, selection criteria, and technical standards in treating this pathology are continuously evolving in response to the available evidence. Therefore, it is essential to establish standardized therapeutic, diagnostic, and care pathways involving different healthcare professions to ensure tailored treatment for individual patients.

As supported by the worldwide literature, diagnostic laparoscopy has become essential in our center for accurately assessing peritoneal disease burden and guiding patients toward the most appropriate treatment options. The direct visualization of peritoneal lesions, particularly in cases of miliary carcinomatosis often undetectable through imaging, is particularly critical in determining the location and the extent of infiltration of peritoneal nodules.

Although we generally consider patients with a PCI below 6 for gastric cancer and below 12 for colorectal cancer to be eligible for cytoreduction, we have included patients with higher PCI scores when the favorable distribution of disease (mainly parietal with sparing of visceral peritoneum) allows for a complete cytoreduction.

Moreover, in order to reduce the rate of sham laparotomies (3.5% vs. 13.6% in patients with and without LPS evaluation, respectively), pre-CRS laparoscopy contributes to surgical planning before the operation. Our collaborative approach, involving vascular, hepatobiliary, gynecological, and urological surgeons, allows us to perform these procedures safely. Despite the technically demanding nature of achieving a high rate of complete cytoreductions (86.6%), we have observed intraoperative complication rates, major postoperative complications, and 30-day mortality rates that are favorable when compared to those reported in the literature [19,20].

However, the risk of major postoperative complications in our experience increases with high PCI scores and significant intraoperative blood loss, as these factors correlate with more complex and extensive surgery. Notably, the learning curve for CRS and HIPEC is substantial but non-standardized due to the complex and heterogeneous surgical techniques required. In a previous study [21], we observed significant improvements in cytoreductive completion rates, postoperative complications, and intraoperative blood loss as the number of procedures performed increased.

In PSM treatment, it is therefore essential to conduct procedures in specialized, high-volume centers.

In our experience, the primary indication for CRS is colorectal cancer (CRS). This is attributed both to the high incidence of this disease and a higher cutoff of PCI for eligibility for cytoreduction, allowing for diagnosis at a stage amenable to surgical treatment. Long-term oncological outcomes are encouraging and align with the existing literature, demonstrating an OS of 52 months and a DFS of 23 months [22,23]. Moreover, as highlighted in the study by Kamada et al. [1], CRS represents a therapeutic option capable of pursuing a curative intent in carefully selected patients. While the therapeutic role of cytoreduction appears established, the utility of hyperthermic intraperitoneal chemotherapy (HIPEC) in CRC remains debated. In the PRODIGE 7 study, the addition of HIPEC with oxaliplatin to CRS did not demonstrate an improvement in survival but was associated with an increase in late postoperative complications [24]. Despite critiques regarding sample size, long data collection periods, selection criteria, and the duration of HIPEC (30 min), the study had a considerable impact on clinical practice, leading several centers, including ours, to shift toward mitomycin-based HIPEC, whose efficacy is currently under evaluation [25].

As a specialized center for PSM, we offer PIPAC to patients who are not candidates for CRS, achieving positive oncological results [26]. Patients with colorectal cancer typically present extensive peritoneal implants that exhibit infiltrative characteristics. As demonstrated in the literature, the penetration of the chemotherapeutic agent into the pathological tissue is limited to a few millimeters [27,28,29]. Therefore, based on the morphological characteristics of carcinomatosis from colorectal cancer, PIPAC has proven to be of limited efficacy. Thanks to an improved understanding of peritoneal carcinomatosis and increased experience of the surgical team, we have progressively refined patient selection for PIPAC, leading to enhanced oncological outcomes.

Gastric cancer with peritoneal metastasis (PM) has a significantly poorer prognosis. However, oncological outcomes achieved with CRS or PIPAC compared to chemotherapy alone are markedly better [30,31]. Patient selection is essential to obtain the best oncological results, with the peritoneal disease burden assessed through the Peritoneal Cancer Index (PCI) score playing a critical role [32,33]. The efficacy of HIPEC remains debated. The first randomized study conducted by Yang et al. in 2011 demonstrated an OS of 11 months in patients undergoing HIPEC compared to 6.5 months in those receiving only CRS [34]. Other randomized studies and meta-analyses have shown survival benefits without differences in morbidity or mortality in the HIPEC group [35,36,37,38]. However, recent findings from the GASTRIPEC-I study indicate that while there was a significant improvement in both progression-free survival and metastasis-free survival, overall survival did not show improvement in the CRS-HIPEC group when compared to patients who underwent only CRS [39]. Further information may be obtained from ongoing randomized studies, including the AIO-FLOT5 trial, which evaluates the effectiveness of chemotherapy alone versus chemotherapy followed by surgical resection in patients with limited metastatic adenocarcinoma of the stomach or esophagogastric junction [40].

In patients with PM from gastric cancer not eligible for CRS, PIPAC is offered, and it is the most common indication at our center. This is primarily due to the difficulty in diagnosing peritoneal carcinomatosis while it is still amenable to surgical treatment. The prognosis in these patients remains rather poor, with an OS of 9 months. However, in our cohort, as well as in other studies reported in the literature [41,42], some patients showed a reduced peritoneal disease extent that was sufficient to permit them to be eligible for CRS. This potential role as a conversion treatment is currently being evaluated in the ongoing prospective, two-arm, randomized multicenter phase III clinical study PIPAC_VEROne [43], which may transform PIPAC from an exclusively palliative treatment into a neoadjuvant therapy.

Consistent with the current scientific evidence and global guidelines regarding the treatment of pseudomyxoma peritonei (PMP), PIPAC has not been performed in these patients, and the only surgical treatment undertaken was CRS. Due to its indolent nature, the diagnosis of PMP is typically made at advanced stages of the disease. The PCI of patients undergoing CRS in our cohort is 21.5, significantly higher than that seen in other histological entities. This is attributed to the low propensity of PMP to cause distant metastases and the limited infiltrative behavior of the carcinomatosis nodules. As a result, even in the presence of extensive peritoneal involvement, lesions can be removed without visceral resections, which usually represents the primary criterion in the case of non-cytoreductive potential.

However, due to the high disease burden, patients with PMP usually undergo particularly prolonged and extensive surgical interventions. This reflects in an acceptable rate of major complications compared to the global literature [44,45,46], although it is higher (17.6%) when compared to carcinomatosis from other primaries such as colorectal cancer (9.8%) or gastric cancer (15.4%). Achieving complete cytoreduction is crucial, as the presence of residual disease is the primary factor influencing long-term outcomes, alongside the execution of HIPEC [47,48,49]. In 2021, an international consensus was published establishing CRS and HIPEC as standard treatments for PMP [50]. In our cohort, HIPEC was consistently performed, except in patients with macroscopic residual disease at the end of the cytoreductive surgical procedure or in those patients indicated for debulking surgery solely for symptomatic relief. The indication for debulking surgery was anecdotal and evaluated exclusively in those patients where symptoms were poorly correlated with intra-abdominal mucin accumulation, but rather attributed to a significant omental cake presentation.

Similarly to PMP, the surgical treatment of mesothelioma is exclusively cytoreductive. Due to the rarity of this disease, our experience is not particularly extensive. However, we observed oncological results consistent with the literature [51,52] in terms of OS and DFS, with respective medians of 43 months and 26 months.

In contrast to the previously nosologically described entities, pancreatic cancer, due to its aggressive local biological behavior and its rapid tendency for distant metastasis, is never a candidate for cytoreduction. In these patients, treatment consisted exclusively of chemotherapeutic regimes, supplemented by PIPAC to increase the intraperitoneal drug concentration and to improve local disease control [53,54,55]. The results regarding survival are encouraging. We observed an OS and DFS of 12 and 4 months, respectively, which are better than those reported for gastric cancer. Similarly, a higher rate of completion of the standard treatment, defined as the performance of at least three PIPAC procedures, was identified in pancreatic cancer if compared both with gastric and colorectal cancer. These findings can be primarily explained by stricter selection criteria for pancreatic cancer regarding the peritoneal disease burden. The median PCI for pancreatic cancer was 8, significantly lower than that for gastric cancer (median PCI of 22) and colorectal cancer (median PCI of 27). Moreover, PIPAC in these patients was initiated during the first lines of treatment when the cells were most chemo responsive, potentially benefiting survival outcomes. The clinical trial NAB-PIPAC is currently underway and may provide further information regarding the efficacy of PIPAC in the treatment of pancreatic carcinomatosis.

CRS and PIPAC have also been performed for non-conventional indications. However, due to the mixture of carcinomatosis from different primary origins included and the heterogeneity of the group, it was deemed impossible to conduct an analysis in terms of oncological outcomes and complications for this patient cohort.

Despite the limitations associated with a non-randomized study and its primarily observational nature, this study represents the real-life experience of a high-volume center and highlights the developments and results achieved over time in the treatment of peritoneal surface malignancies. Furthermore, in the absence of definitive evidence regarding treatment efficacy for peritoneal carcinomatosis and well-defined guidelines, data from large retrospective series can still be valuable in guiding oncological and surgical strategies for these patients.

## 5. Conclusions

The integration of CRS, HIPEC, and PIPAC with oncological therapies has shifted the paradigm of peritoneal surface malignancies from a palliative condition to a potentially curable disease. Diagnostic laparoscopy and a multidisciplinary pre-operative assessment are essential for patient selection and treatment planning. Multidisciplinary evaluation conducted by highly specialized clinicians and surgeons from high-volume centers is crucial to deliver tailored treatment strategies, optimizing oncological outcomes while maintaining patients’ quality of life.

## Figures and Tables

**Table 1:** Baseline characteristics and surgical details in patients who underwent cytoreduction (**A**) and PIPAC (**B**).

(**A**)		(**B**)	
** Variable**	**CRS**	** Variable**	**PIPAC**
	**389 Patients**		**183 Patients**
** Age median (Min–Max)**	60 (26–86)	** Age median (Min–Max)**	62 (34–81)
**Gender (%)**		**Gender**	
** ** Male	139 (35.73)	** ** male	76 (41.53)
** ** Female	250 (64.27)	** ** female	107 (58.47)
**BMI median (Min-Max)**	24 (16–42)	**BMI median (Min-Max)**	22.3 (16.2–38.1)
**ECOG n (%)**		**ECOG n (%)**	
** ** Grade 0	224 (57.59)	** ** Grade 0	52 (28.41)
** ** Grade ≥ 1	165 (42.41)	** ** Grade ≥ 1	131 (71.59)
**Primary Tumor n (%)**		**Primary Tumor n (%)**	
** ** Colorectal Cancer	204 (52.44)	** ** Gastric Cancer	69 (38)
** ** Gastric Cancer	39 (10)	** ** Colorectal Cancer	31 (16.9)
** ** PMP	104 (26.73)	** ** Hepato-billiaric cancer	14 (8.2)
** ** Mesothelioma	27 (6.94)	** ** Pancreatic Cancer	56 (30.5)
** ** Other	15 (3.85)	** ** Other Primary Tumor	12 (6.5)
**Timing Carcinosis (%)**		**Timing Carcinosis n (%)**	
** ** Synchronous	264 (67.86)	** ** Synchronous	101 (55.19)
** ** Metachronous	125 (32.14)	** ** Metachronous	82 (44.81)
**Treatment of Recurrence n(%)**	48 (12.33)	**PCI median (Min–Max)**	18 (2–39)
**PCI median (Min–Max)**	8 (1–35)	**Extraperitoneal Metastases at PIPAC1 n (%)**	24 (13.11)
**Median of surgical procedure**	5 (2–12)	** ** Hepatic	13 (7.10)
** ** Right colectomy	127 (32.64)	** ** Pulmonary	6 (3.27)
** ** Left colectomy	29 (7.45)	** ** Bone	3 (1.64)
** ** Rectal resection	170 (43.70)	** ** Hepatic + Bone	2 (1.09)
** ** Pelvic Peritonectomy	268 (68.89)	**Chemotherapy Drug per procedures n (%)**	370
** ** Diaphragmatic peritonectomy	178 (45.76)	** ** Oxaliplatin	74 (20)
** ** Mesenteric cytoreduction	86 (22.11)	** ** Cisplatin + Doxorubicin	223 (60.27)
** ** Hysterectomy/oophorectomy	172 (44.22)	** ** Nab-Paclitaxel	73 (19.73)
** ** Omentectomy	325 (83.54)	**Concomitant procedure n (%)**	14 (3.78)
** ** Gastric resection	56 (14.39)	** ** Hystero-annexectomy	1 (0.27)
** ** Small bowel resection	104 (26.73)	** ** Bilateral annexectomy	6 (1.62)
** ** Segmental liver resection	29 (7.45)	** ** Monolateral annexectomy	3 (0.81)
** ** Splenectomy	70 (17.99)	** ** Bowell resection	2 (0.54)
** ** Ostomy	126 (32.39)	** ** Wall nodule removal	2 (0.54)

**Table 2:** Intraoperative outcomes in cytoreductive surgery (CRS), pressurized intraperitoneal aerosol chemotherapy (PIPAC), and diagnostic laparoscopy.

Variable	CRS	PIPAC	Diagnostic LPS
	389 Procedures	370 Procedures	354 Patients
**Median Operative time (Min–Max)**	417 (158–1005)	125 (66–271)	46 (33–171)
**Blood loos > 500**			
** ** Yes	75 (19.28)	2 (0.54)	-
** ** No	314 (80.2)	368 (99.46)	354 (100)
**Intra-operative complication**			
** ** Yes	54 (13.88)	2 (0.54)	1 (0.28)
** ** No	335 (86.12)	368 (99.46)	353 (99.82)
**Types of Intraoperative complication**			
Bowel perforation	3 (0.77)	2 (0.54)	1 (0.28)
Bladder injury	11 (2.82)	-	-
Vaginal injury	1 (0.26)	-	-
Ureteral injury	2 (0.51)	-	-
Diaphragmatic laceration	29 (7.45)	-	-
Duodenal perforation	1 (0.26)	-	-
Gastric perforation	1 (0.26)	-	-
Vascular injury			
** ** Common iliac vein	1 (0.26)	-	-
** ** Internal iliac vein	1 (0.26	-	-
** ** Supra-hepatic vein	1 (0.26)	-	-
** ** Inferior mesenteric artery	1 (0.26)	-	-
** ** Caval vein	2 (0.51)	-	-

**Table 3:** Postoperative complication in CRS, PIPAC, and diagnostic laparoscopy.

Variable	CRS	PIPAC	Diagnostic LPS
	389 Procedures	370 Procedures	354 Patients
**Postoperative complication**			
** ** Yes	155 (39.8)	58 (15.67)	35 (9.88)
** ** No	234 (60.2)	312 (84.32)	319 (90.11)
**Clavien–Dindo grade**			
** ** I	32 (8.22)	22 (5.95)	7 (1.9)
** ** II	81 (20.82)	28 (7.57)	18 (5.08)
** ** IIIa	26 (6.68)	5 (1.35)	4 (1.13)
** ** IIIb	17 (4.37)	2 (0.54)	2 (0.56)
** ** IV	6 (1.54)	1 (0.27)	-
**Major Postoperative complications (III-IV)**	49 (12.59)	8 (3.16)	6 (1.69)
** ** Abdominal collection	14 (3.59)	2 (0.54)	2 (0.56)
** ** Delay emptying gastric syndrome	2 (0.51)	-	-
** ** Pleura effusion	7 (1.79)	3 (0.81)	2 (0.56)
** ** Pneumothorax	3 (0.77)	-	-
** ** Abdominal bleeding	8 (2.06)	2 (0.54)	1 (0.28)
** ** Anastomotic leak	6 (1.54)	-	-
** ** Evisceration	3 (0.77)	-	1 (0.28)
** ** Hemorrhagic shock	3 (0.77)	-	-
** ** Septic shock	2 (0.51)	-	-
** ** Cardiac failure	1 (2.08)	1 (12.5)	-
**30-day mortality**	5 (1.28)	4 (1.08)	-

**Table 4:** Clinical, surgical, and oncological features associated with major postoperative complications (CD ≥ 3). Univariate and multivariate analysis.

Variables	Univariate Analysis			Multivariate Analysis		
	OR	95% CI	*p*-Value	OR	95% CI	*p*-Value
**Age**	1.016	[0.999–1.043]	0.257			
**Gender**						
** ** Male	1.113	[0.603–2.055]	0.732			
** ** Female						
**BMI (Body Mass Index)**	1.128	[0.501–2.652]	0.833			
** ** <18						
** ** 19–29						
** ** >30						
**ECOG > 1**						
** ** Yes	2.625	[0.639–10.810]	0.182			
** ** No						
**ASA > 3**						
** ** Yes	1.22	[0.493–3.017]	0.667			
** ** No						
**Primary Tumor**						
** ** Colorectal Cancer	0.436	[0.234–0.812]	0.009	1.181	[0.421–3.311]	0.752
** ** Gastric cancer	1.459	[0.418–2.680]	0.904			
** ** PMP	1.731	[0.935–3.205]	0.81			
** ** Mesothelioma	1.288	[0.471–4.586]	0.498			
** ** Other	1.291	[0.731–3.042]	0.272			
**Timing of carcinosis**						
** ** Synchronous	2.047	[1.013–4.134]	0.046	1.06	[0.384–2.924]	0.911
** ** Metachronous						
**PCI**	1.075	[1.034–1.117]	0.0001	1.059	[1.014–1.107]	**0.01**
**Prior surgery**						
** ** Yes	0.92	[0.350–1.099]	0.207			
** ** No						
**Surgical procedure**						
** ** Hepatectomy	1.479	[0.569–3.845]	0.422			
** ** Splenectomy	1.895	[1.043–3.442]	0.036	0.503	[0,165–1.539]	0.229
** ** Gastric resection	2.43	[1.180–5.005]	0.016	5.251	[1.889–14.593]	**0.001**
** ** Colic resection	1.86	[0.952–3.632]	0.069			
** ** Rectal resection	1.191	[0.852–1.667]	0.306			
** ** Bowell resection	0.788	[0.410–1.518]	0.477			
** ** Istero-annessicetomy	0.967	[0.535–1.747]	0.911			
** ** Diaphragmatic peritonectomy	2.879	[1.567–5.287]	0.001	1.879	[0.676–5.223]	0,226
** ** Pelvic peritonectomy	2.464	[1.158–5.242]	0.019	1–559	[0.458–5.307]	0.477
**Blood loss > 500 mL**						
** ** Yes	3.48	[1.865–6.492]	0.001	4.286	[1.780–10.317]	**0.001**
** ** No						
**Number of Anastomosis**	1.579	[1.103–2.257]	0.013	1.075	[0.638–1.811]	0.789
**HIPEC**						
** ** Yes	0.879	[0.306–1.095]	0.198			
** ** No						
**Pre-operative Chemotherapy**						
** ** Yes	0.962	[0.689–1.556]	0.283			
** ** No						
**Intra-operative Complications**						
** ** Yes	1.327	[0.690–2.555]	0.397			
** ** No						

Bold values indicate statistical significance.

## Data Availability

The raw data supporting the conclusions of this article will be made available by the authors on request.
